# The role of altered microRNA expression in premalignant and malignant head and neck lesions with epithelial origin

**DOI:** 10.1002/hsr2.921

**Published:** 2022-11-06

**Authors:** Alieh Farshbaf, Farnaz Mohajertehran, Amirhossein Sahebkar, Yasaman Garmei, Parisa Sabbagh, Nooshin Mohtasham

**Affiliations:** ^1^ Dental Research Center Mashhad University of Medical Sciences Mashhad Iran; ^2^ Department of Oral and Maxillofacial Pathology, School of Dentistry Mashhad University of Medical Sciences Mashhad Iran; ^3^ Biotechnology Research Center, Pharmaceutical Technology Institute Mashhad University of Medical Sciences Mashhad Iran; ^4^ Applied Biomedical Research Center Mashhad University of Medical Sciences Mashhad Iran; ^5^ Department of Biology, Faculty of Science Sistan and Balouchestan University Zahedan Iran

**Keywords:** biomarker, microRNA, oral cavity, oral potentially malignant disorders

## Abstract

**Background and Aims:**

The premalignant lesions of the oral cavity carry a risk of transformation to malignancy. Hence, early diagnosis followed by timely intervention remarkably affects the prognosis of patients. During tumorigenesis, particular microRNAs (miRNAs) show altered expressions and because of their post transcriptionally regulatory role could provide favorable diagnostic, therapeutic, or prognostic values in head and neck cancers.

**Methods:**

In this review, we have demonstrated diagnostic, prognostic, and potential therapeutic roles of some miRNAs associated with oral premalignant and malignant lesions based on previous validate studies.

**Results:**

It is previously documented that dysregulation of miRNAs contributes to cancer development and progression. MiRNAs could be tumor suppressors that normally suppress cell proliferation, differentiation, and apoptosis or play as oncogenes that improved tumorigenesis process. Altered expression of miRNAs has also been reported in premalignant oral epithelial lesions such as leukoplakia, oral submucous fibrosis, oral lichen planus and some malignant carcinoma like oral squamous cell, verrucous, spindle cell, Merkel cell carcinoma and basal cell.

**Conclusion:**

Some of miRNAs could be new therapeutic candidates in miRNA‐based target gene therapy. Although more investigations are required to identify the most favorable miRNA candidate, altered expression of some miRNAs could be used as biomarkers in premalignant lesions and oral cancers with high sensitivity and specificity.

## 1 INTRODUCTION

The in‐situ lesions of the oral cavity are considered premalignant with a variant tendency to develop into malignant tumors. Also, oral malignant disorders are locally invasive and carry a risk of distant metastasis. Hence, early diagnosis followed by timely intervention remarkably affects the prognosis of these patients.^1^ The oral cavity in‐situ lesions with epithelial origin include proliferative verrucous leukoplakia (PVL), erythroplakia, oral submucous fibrosis, erythroleukoplakia, granular leukoplakia, laryngeal keratosis, actinic cheilosis, and lichen planus (LP). And also oral and maxillofacial malignant tumors are squamous cell carcinoma (SCC), verrucous carcinoma, oropharyngeal carcinoma, spindle cell carcinoma (sarcomatoid SCC, polypoid SCC, carcinosarcoma, pseudosarcoma), adenosquamous carcinoma, basaloid SCC, carcinoma of the maxillary sinus, sinonasal undifferentiated carcinoma, nasopharyngeal carcinoma, basal cell carcinoma, Merkle cell carcinoma and melanoma.^2^ Clinically suspected lesions are confirmed by histopathological examination. During the evaluation of cancerous lesions, the expression of some molecular biomarkers could facilitate early diagnosis.

**Figure 1:**
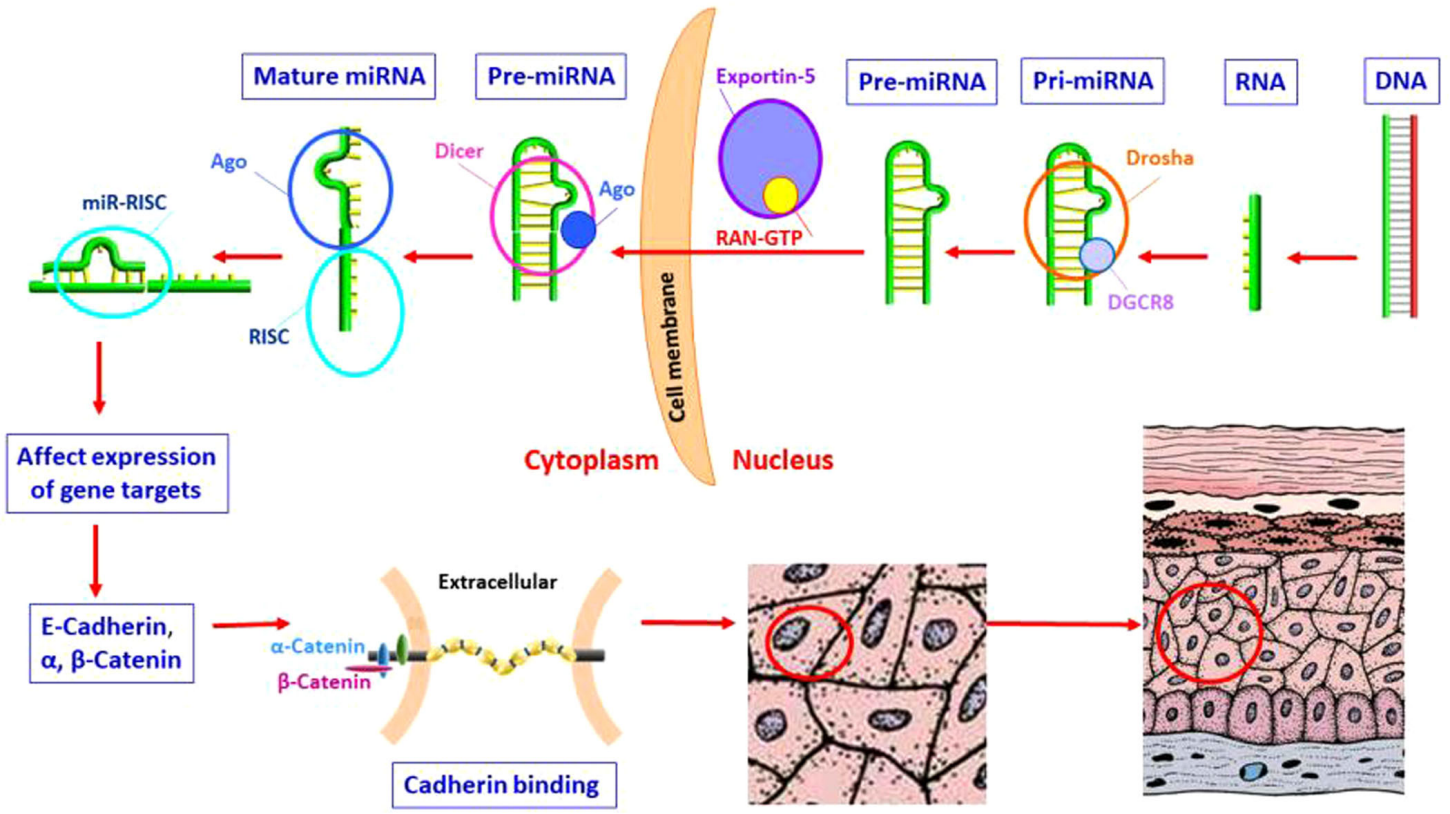
The process of miRNA synthesis in oral epithelial mucosal that some of them affect extracellular binding proteins. miRNAs, microRNAs.

The microRNAs (miRNAs) are a class of noncoding RNAs with 21–23 nucleotides in length which are naturally found in the form of hairpin structures that regulate gene expression by silencing transcription or inhibition of translation.^3^, ^4^ They usually target 3′ untranslated region (3′ UTR) and to a lesser extent to 5′ UTR to accelerate degradation of certain mRNAs. Also, miRNAs mediate cellular proliferation, differentiation, and apoptosis, among others. The miRNA synthesis process is schematically demonstrated in Figure 1 that affects extracellular binding proteins and oral epithelial mucosal. MiRNAs are involved in various physiological processes such as differentiation and development, and numerous studies have demonstrated their significant role in several diseases. In human dental tissues, miRNAs may have important functions related to periodontal disease, tooth movement and eruption, dental pulp physiology and pathology, dental cell differentiation, enamel mineralization, and cancerous lesions.^5^ Specific miRNA expression profiles have been reported to be predictive of certain clinical outcomes in the oral cavity and could be used as biomarkers for diagnostic and prognostic purposes.^5^ Previous studies have highlighted the potential diagnostic role of miRNAs in the management of oral diseases and cancerous lesions.^3^, ^6^, ^7^, ^8^, ^9^, ^10^, ^11^ In this review, we have described some alternations in the expression profile of miRNAs that can apply as novel biomarkers in disease control and predicted survival in head and neck premalignant and malignant lesions. The aim of this study is a description of the remarkable role of some miRNAs as diagnostic, prognostic biomarkers, and a potential candidate in therapeutic approaches of different head and neck lesions. In this manner, the miRNA types and their target genes with function are mentioned to highlight their alternation involved in lesion pathogenesis.

## 2 THE PREMALIGNANT ORAL EPITHELIAL LESIONS

### 2.1 Leukoplakia (leukokeratosis, erythroleukoplakia, erythroplakia)

Leukoplakia is a precancerous lesion that has histopathologic features, including hyperkeratosis with or without acanthosis. Some leucoplakias show epithelial atrophy. Epithelial dysplasia or carcinoma is found in about 5%–25% of oral leukoplakia. Dysplastic changes begin in the basal and supra basal epithelium. They mostly located in the oral cavity after the age of 40. It's clinical appearance varies from persistent white plaques, thin, smooth leukoplakia to advanced erythroplakia.^2^, ^12^ It is assumed that consumption of tobacco, alcohol, sanguinaria, exposure to ultraviolet radiation, certain infections, and trauma may predispose its formation. Cytogenetic and molecular changes associated with leukoplakia include loss of heterozygosity (LOH), microsatellite instability, increased telomerase activity, and upregulation of p53, p16, EFGR, MMR, and VEGF.^13^ Moreover, altered expressions of some miRNAs such as miR‐450b‐5p, miR‐21, and miR‑1 have also been reported in the pathogenesis of leukoplakia, and associated with other cancers (Table 1). All these findings may apply as diagnostic, prognostic, and future therapeutic markers.^18^

**Table 1:** Some studies related to alternation in miRNA expression for Leukoplakia

miRNA	The mRNA target gene of miRs	miRNA function	Pathogenesis associated with miRNA alteration	Reference
miR‐450b‐5p, miR‐129‐5p, miR‐296‐5p	Transcriptional regulation, estrogen signaling pathway, p53 signaling pathway, and RIG‐I‐like receptor signaling pathway	Discrimination of OLK from OLK‐OSCC	miR‐129‐5p downregulated in medullary thyroid carcinoma and lung cancer cell lines and lung cancer tissues miR‐296 overexpressed in prostate cancer miR‐296‐5p decreased in breast cancer miR‐450b‐5p upregulated in colorectal cancer	14
miR‐21, miR‐31	HIF	miR‐31: it mediates oncogenesis by targeting a molecule that inhibits hypoxia inducing‐factor in oral cancer and helps the cells to escape from apoptosis and chemotherapy	miR‐31 overexpressed in lung cancer	15
miR‑191			miR‑191 is expressed in breast cancer, prostate cancer, colon cancer, and oral cavity	16
miR‐150‐5p/miR‐222‐3p		miR‐222‐3p: associated with tumor progression and lymph node metastasis		17

Abbreviation: miRNAs, microRNAs.

### 2.2 Oral submucous fibrosis

Oral submucous fibrosis (OSMF) is a precancerous lesion characterized by chronic and progressive scar formation in oral mucosa likely due to disrupted collagen metabolism and consumption of areca nut (betel quid), nutritional deficiency, or tobacco smoking. The disease symptoms include trismus, stomatitis, and intolerance of spicy foods. Histopathologic features are juxta epithelial and submucosal deposition of densely collagenized, hypovascular connective tissue along with the accumulation of chronic inflammatory cells. Mild cases are treated with intralesional corticosteroids, whereas moderate to severe cases require surgical splitting or excision of fibrous bands.^19^ It is shown that expression of some molecular biomarkers such as miR‐2, miR‐31, miR‐203, and miR‐1246 changes following the formation of OSMF, which could be of great significance in the management of this lesion (Table 2).

**Table 2:** List of some miRNA associated with oral submucous fibrosis pathogenesis

miRNA	The mRNA target gene of miRs	miRNA function	Pathogenesis associated with miRNA alteration	Reference
miR‐1246	Type I collagen α1 (COL1A1), 2 (COL1A2), and 3 (COL1A3)	miR‐1246 activate Wnt/β‐catenin signaling, and TGF‐β1‐induced type I collagen expression, also contributes to fibrogenesis in the oral cavity	Liver, breast, and colon cancers	20
miR‐203	miR‐203 negatively regulated SFRP4 and positively regulated TM4SF1 in oral submucous fibrosis	miR‐203 downregulated N‐cadherin, vimentin, and cell proliferation also increased CK19 and E‐cadherin proteins. It inhibits arecoline‐induced epithelial‐mesenchymal transition	Chronic inflammatory skin diseases like psoriasis and atopic eczema	21
miR‐21	Apoptotic marker: tropomyosin1 and phosphatase tensin homolog	miR‐21 overexpression inversely associated with levels of tropomyosin1 and phosphatase tensin homolog and a worse average survival rate of carcinomas with a shorter disease‐free period (prognostic value)	Overexpressed in six solid cancers such as lung, breast, stomach, prostate, colon, and pancreas	22
miR‐31	CXCL12	Significant upregulation	Promotes cancer development during regulation of cell transformation, proliferation, cell cycle, apoptosis, and metastasis. It detected in malignancies of lung cells and may play a vital role in oral cancer development	23

Abbreviation: miRNAs, microRNAs.

### 2.3 Oral lichen planus (OLP)

Lichen planus is a chronic inflammatory disease that may affect skin, genitalia, nails, and oral mucosa. Erosive lichen planus (ELP) is a variant of LP with painful ulcerations that is formed by autoimmune damage of the basal cell layer. The most common lesions of LP are purple papules with irregular borders and a pattern of white lines known as Wickham striae on their surface. Lesions of ELP present as atrophic, erythematous with central ulceration.^2^ Histopathologic features include a saw‐toothed rete ridge, hydropic degeneration of basal cell layer, and keratinocyte degeneration known as colloid bodies. However, the immunopathology characteristics of LP are not specific. There is also a potential malignant transformation of ELP to squamous cell carcinoma (SCC). Some cases present dysplastic leukoplakia, which is a secondary lichenoid inflammatory infiltrate that resembles OLP.^24^ There is an association between hepatitis C and OLP particularly in specific populations of the Mediterranean region that highlights genetic profile and geographical distribution of LP.^25^ An overexpression profile in some miRNAs like miRNA‐146a/miRNA‐155 and miR‐150‐5p/miR‐222‐3p stimulate Th1 response in OLP, improve autoimmune disease which supports the contributory role of miRNAs in identification of genes involved in development of OLP (Table 3). Of note, OLP, particularly erosive‐atrophic type, has been widely confirmed as a potentially malignant disorder. On the other hand, OLP and SCC are common oral lesions that may have separate causes.^36^ There are other premalignant oral epithelial lesions with limited research which demonstrate altered miRNA expression including smokeless tobacco use, smokeless tobacco keratosis (snuff pouch; snuff dipper's lesion; tobacco pouch keratosis; spit tobacco keratosis).^2^

**Table 3:** List of alternation in expression of some miRNA in oral lichen planus

miRNA	The mRNA target gene of miRs	miRNA function	Pathogenesis associated with miRNA alteration	Reference
miR‐26a/b		miR‐26a/b inhibited apoptosis with PKCδ and suppressed Th1‐related cytokines secretion	miR‐26a/b down regulated in metabolic disease and cancer	26
miR‐146a, miR‐155	Both target transcripts that affect the differentiation of CD4^+^ Tcells following Th1 or Th2 responses	miR‐146a and miR‐155 increased Th1 response in OLP in response to an unknown autoantigen, providing the imbalance of Th1/Th2 cytokines toward Th1immunity (IFN‐γ production) which stimulates local immune responses against an antigen in disease progression.	Alternation in expression of miR‐146a and miR‐155 in chronic inflammatory situations such as periodontal diseases, rheumatoid arthritis, and Sjogren's syndrome	27, 28
miR‐155	Target genes associated with inflammation	miR‐155 associated with inflammation, immune responses, tumor development, functioning mainly as a tumor‐promoting factor	Overexpression of miR‐155 in breast, colon, cervical, and lung cancer	29
miR‐27b‐3p	cypd, grem1, litaf, tmsb10, itga5, and mesdc1	Downregulation of miR‐27b‐3p inhibited epithelial keratinocytes apoptosis in OLP with upregulation of cyclophilin D expression. Then increased Bcl2 which suppressed cas9/3 activation and cyt C release.	Breast cancer	30
miR‐21, miR‐125b, miR‐203, miR15b	p53, p63	miR‐21 has an antiapoptotic role with increasing cell proliferation miR‐125b suppressing p53 and TNF‐α pathway28 mir15b improving induction of regulatory T‐cells (Immune regulation) 14	miR‐21: breast, lung and colon cancers miR‐125b: Psoriasis, prostate cancer miR‐15b: breast cancer miR‐203: chronic inflammatory skin diseases such as psoriasis and atopic eczema	31, 32
miR‐138	cyclin D1 (CCND1)	The precursor of miR‐138 is expressed ubiquitously, the mature product is found only in specific cell types	HNSCC, nasopharyngeal carcinoma (NPC) Brain cancer	33
miR‐802	Bcl‐2	It regulates apoptosis of cancer cells	Gastric cancer, tongue squamous cell carcinoma, epithelialmesenchymal transition, and liver	34
miR‑122, miR‑199a‑3p	miR‐122: Akt, LC3B, IGF1 miR‑199a‑3p: mTOR, LC3B	miR‐122: tumor suppressor and has a critical role in inhibiting the tumorigenesis and angiogenesis miR‑199a‑3p: can modulate cell proliferation by inhibiting mTOR expression	miR‐122: breast cancer	35

Abbreviations: miRNAs, microRNAs; OLP, oral lichen planus.

## 3 THE MALIGNANT ORAL EPITHELIAL LESIONS

### 3.1 Oral squamous cell carcinoma (OSCC)

Squamous cell carcinoma represents more than 90%of oral malignancies. Risk factors for malignant transformation include tobacco smoking, alcohol consumption, exposure to UV radiation, occupational exposure to solvent and heavy metal dust, environmental pollutants, iron deficiency, and HPV infection.^36^, ^37^, ^38^ As seen in Table 4, the regulation of some biomarkers such as miRNA is significantly disrupted in OSCC. Also, the profile of gene expression changes during OSCC process.^42^, ^43^ In Table 4, we mentioned some miRNAs like miR‐423‐5p, miR‐146a, miR‑21, and target genes that their expression alters significantly in oral squamous cell carcinoma.

**Table 4:** List of alternation in expression of some miRNA in OSCC

miRNA	The mRNA target gene of miRs	miRNA function	Pathogenesis associated with miRNA alteration	Reference
miR3162, miR‐3651, miR‐494, miR‐3162 let‐7d	miR‐186 target cell‐cycle regulation genes	miR‐186 regulate apoptotic response, and is an important marker for diagnosis, prognosis, and therapy in OSCC. When overexpressed in cancer tissue play as an anti‐invasion target for therapeutic miR‐494 as a tumor suppressor induce cell‐cycle arrest, cell senescence, apoptosis, and repress cell proliferation miR‐3651 and miR‐3162: there are not enough investigation in cancer development and progression let‐7d play role in embryonic development and tumorigenesis prevention	miR‐186 downregulated in non‐small cell lung cancer, esophageal cancer, and lung adenocarcinoma. let‐7d downregulated in tissues of several types of tumors including HNSCC.	39, 40
miR‑21, miR‑191		miR‑21 is an oncogenic miR.	miR‑21: it has been significantly upregulated in oral squamous cell carcinoma and premalignant lesions miR‑191 is overexpressed in breast cancer, prostate cancer, colon cancer, and oral cavity	16
miR‑191, miR‐146a	miR‐191: CCAAT, TIMP3	miR‐146a: enhance tumorigenesis and also it associates with downregulation of the IL‐1 receptor associated with kinase 1 (IRAK1), TNF receptor‐associated factor 6 (TRAF6), and NUMB endocytic adapter protein (NUMB)	miR‐191: colorectal cancer, breast prostate cancer, and acute myeloid leukemia miR‐146a deregulation has been found in oral cancer	41
miR‐150‐5p/miR‐423‐5p	miR‐150‐5p: mTOR‐HIF‐1alpha, VEGF‐Apathway miR‐423‐5p: TTN‐AS1	Both overexpressed and can apply as predictor for tumor progression	Both correlated with clinical stage, lymph node metastasis status, and stage	17

Abbreviations: HNSCC, head and neck squamous cell carcinoma; miRNAs, microRNAs; OSCC, oral squamous cell carcinoma.

### 3.2 Verrucous carcinoma (snuff dipper's cancer; ackerman's tumor)

Verrucous carcinoma, a low‐grade SCC that comprises less than 16% of oral cancers, is related to smokeless tobacco use and presents with verruciform surface projections. It may develop from high‐risk precancer, PVL and has a better prognosis than SCC with no potential for lymph node or distant metastases. In addition to some immunopathologic markers, there are some molecular biomarkers for diagnosis of verrucous carcinoma namely miRNA‐195 which downregulates the expression of CDK6 gene.^44^ Alternation in the expression of miRNA‐195 provides a double edge role as a tumor suppressor or oncogene factor that affects multiple pathways including proliferation, metastasis, and apoptosis. In this manner, it involved in a broad range of cancers such as promoting tumorigenesis in gastric, hepatocellular, esophageal, brain, bone cancer, lung, skin, prostate, and cervical cancers.^45^

### 3.3 Spindle cell carcinoma

Spindle cell carcinoma is a rare OSCC characterized by dysplastic epithelium and invasive spindle elements which are strongly associated with alcohol consumption and tobacco smoking. Spindle cell carcinoma of aerodigestive tract is found in the upper aerodigestive tract particularly in larynx, alveolar mucosa, tongue, buccal mucosa, and lower lip. Previously, dysregulation of miR‐200 family and miR‐205 target classic (E‐ and N‐cadherins) which mediate epithelial‐mesenchymal transition were reported in pancreatic cancer. These miRNAs are also found to be downregulated in spindle cell carcinoma.^46^

### 3.4 Basal cell carcinoma (basal cell epithelioma, rodent ulcer)

Basal cell carcinoma (BCC) is the most common skin cancer that frequently arises from basal cells of the epithelium layer in the head and neck region. The main known risk factor is UV radiation but mutations in melanocortin 1 receptor (MC1R*)* gene or hedgehog pathway genes (e.g., patched (PTCH)) and activation of smoothened (SMO) also enhance susceptibility to BCC.^47^ Untreated lesions manifest as rodent ulcers. Subtypes include nodulocystic, pigmented, keratotic, adenoid, superficial, infiltrative, morphea form, and micronodular. In Table 5, we have listed alternations in the expression of miRNAs found in BCC.

**Table 5:** Some of miRNAs involved in pathogenesis of basal cell carcinoma

miRNA	The mRNA target gene of miRs	miRNA function	Pathogenesis associated with miRNA alteration	Reference
miR‐451a	TBX1	miR‐451a significantly suppressed cell growth in G1 cell cycle arrest	Autism spectrum disorders and neuronal injury	48
miR‐145‐5p	MDM2 and p53	Upregulating of miR‐145‐5p affected by p53, and posttranscriptional upregulated the miR‐143‐145 cluster with a MDM2‐p53 feedback loop. downregulation of miR‐145‐5p observed in p53 mutated cancers.	Vascular disease and diffuse large B‐Cell lymphoma	49
miR‐203, miR‐495, miR‐381, miR‐200a, and miR‐30e	ING3, VEGFA, TP63, MMP11, NRP1, HIF1A, APC, PTCH1	Upregulating ofTP63, NRP1, MMP11, PTCH1 downregulation ofING3, APC, HIF1A, VEGFA	miR‐203: skin diseases such as psoriasis and certain types of cancers	50

Abbreviation: miRNAs, microRNAs.

### 3.5 Merkel cell carcinoma

Merkel cell carcinoma is a rare, rapidly progressive neuroendocrine tumor that typically manifests as a painless nodule on the head or neck. Risk factors include exposure to UV radiation, immunosuppressive therapy (e.g. transplant recipients), malignancy, HIV infection, adults older than 70 years. In more than 80% of patients, a trace of a novel Merkel cell polyomavirus genome is reported. Immunohistochemistry for neuroendocrine markers (chromogranin A, synaptophysin, neuron‐specific enolase, and CD56) and neurofilament implies Merkel cell origin whereas, nonendocrine epithelial and sarcomatous markers indicate pluripotent stem cells from epidermal or dermal origin.^2^ Merkel cell carcinoma can be differentiated from metastatic small cell carcinoma of the lung by lack of immunoreactivity for thyroid transcription factor 1 (TTF‐1). About 25% of cases develop additional malignancies including SCC of the skin, hematologic malignancies, or adenocarcinomas of the breast or ovaries. Hence, patients with Merkel cell carcinoma should be monitored regularly.^2^ MiR‐34a, which is related to p53‐dependent apoptosis promotion and cell cycle regulation and is involved in the pathogenesis of Merkel cell carcinoma, is listed in Table 6. However, there are few reports on the immunobiological markers in extremely rare variants of SCC, including papillary squamous cell carcinoma (PSCC) and adenoid (acantholytic) squamous cell carcinoma (AdSCC). But according to the information from the reliable online databases, the articles related to the expression of specific microRNA and PSCC and ASCC lesions as biomarkers has not been published.

**Table 6:** Some of miRNAs involved in pathogenesis of Merkel cell carcinoma

miRNA	The mRNA target gene of miRs	miRNA function	Pathogenesis associated with miRNA alteration	Reference
miR‐34a	SIRT1, bcl‐2	miR‐34a mediated with p53‐dependent apoptosis promotion and cell cycle regulation	HPV‐induced cervical cancer, hepatocellular carcinoma	51
miR‐375	Target genes involved in Hippo‐ and epithelial to mesenchymal transition (EMT) signaling pathways, neuroendocrine differentiation	miR‐375may serve intercellular rather than intracellular signaling in MCC	Low expressionin gastric, pancreatic, colon, and liver cancer, and high expression in medullarythyroid carcinoma, andprostate cancer	52, 53

Abbreviation: miRNAs, microRNAs.

## 4 THE RELATIVE COMMON HEAD AND NECK MALIGNANT LESIONS

### 4.1 Carcinoma of maxillary sinus

Carcinoma of maxillary sinus or antrum is an uncommon malignancy with unknown etiology that is associated with improved survival rates among HPV‐positive groups. Although most lesions are squamous cell carcinomas, the other subtypes include sinonasal adenocarcinoma, sinonasal undifferentiated carcinoma (SNUC), neuroendocrine carcinoma, and salivary gland adenocarcinoma. Patients present in advanced stages when maxillary sinus carcinoma is diagnosed hence contributing to its poor prognosis.^54^ The miRNA expression profile of this tumor is demonstrated in Table 7.

**Table 7:** Some of miRNAs involved in pathogenesis of relative common head and neck malignant lesions

Lesion type	miRNA	The mRNA target gene of miRs	miRNA function	Pathogenesis associated with miRNA alteration	Reference
Carcinoma of the maxillary sinus	miR‐204	EphA7	Tumor suppressor		55
Nasopharyngeal carcinoma	miR‐150	EMT	miR‐150 can modulate the epithelial‐mesenchymal‐transition property in NPC/HK‐1 cells and provide motility and invasion. It plays a pivotal role in NPC tumorigenesis with a potential biomarker.	Myasthenia Gravis Rheumatoid Arthritis Gastric Cancer	56
	miR‐1, miR let‐7, miR‐9, miR‐26a, miR‐29c, miR‐98, miR‐124, miR‐138, miR‐184, miR‐200, miR‐204, miR‐216b, miR‐375, miR‐451 miR‐10b miR‐18a miR‐18b miR‐21 miR‐30a, miR‐93 miR‐141 miR‐144 miR‐149 miR‐155 miR‐205 miR‐214 miR‐378 miR‐421 miR‐663	miR‐1: PTMA miRNA let‐7: c‐Myc, EZH2 miR‐9: CXCR4 miR‐26a: EZH2, c‐Myc miR‐29c: TIAM1 miR‐98: EZH2 miR‐124: Foxq1 miR‐138: CCND1 miR‐184: BCL2, c‐Myc miR‐200: ZEB2, CTNNB1, Notch1 miR‐204: Stat‐3, CDC42 miR‐216b: PKCa, K‐Ras miR‐375: MTDH miR‐451: MIF miR‐10b: MMP‐9 miR‐18a: Dicer1, c‐Jun, c‐Myc miR‐18b: CTGF miR‐21: BCL2 miR‐30a: E‐cadherin miR‐93: TGFBR2 miR‐141: BRD3, PTEN, SPLUNC1 miR‐144: PTEN miR‐149: E‐cadherin miR‐155: JMJD1A, BACH1 miR‐205: PTEN miR‐214: LTF, Bim miR‐378: TOB2 miR‐421: FOXO4 miR‐663: p21	miR‐1: Induces carcinoma cell apoptosis miR let‐7: Inhibits cell proliferation and induces cell apoptosis miR‐9: Regulates proliferation, EMT, invasion, metastasis, apoptosis, and tumor angiogenesis miR‐26a: Suppresses cell proliferation and colony formation miR‐29c: Inhibits cell migration and invasion miR‐98: Inhibits relapse miR‐124: Inhibits cell growth, migration, and invasion miR‐138: Suppresses cell proliferation and colony formation miR‐184: Suppresses cell proliferation miR‐200: Regulates EMT, migration, and invasion miR‐204: Regulates invasion miR‐216b: Suppresses proliferation and invasion miR‐375: Suppresses relapse miR‐451: Regulates NPC cell growth and invasion miR‐10b: Promotes mobility and invasion miR‐18a: lymph node metastasis miR‐18b: Promotes cell growth miR‐21: Promotes migration and proliferation miR‐30a: Increases the capability of metastasis and invasion miR‐93: Promotes cell proliferation, invasion, and metastasis miR‐141: Promotes cell growth, migration, and invasion miR‐144: Promotes migration and invasion miR‐149: Promotes mobility and invasion miR‐155: Stimulates cell proliferation, colony formation, cell migration, and invasion miR‐205: Attenuates cell apoptosis postirradiation miR‐214: Promotes NPC cell proliferation, invasion, and metastasis miR‐378: Promotes cell proliferation, colony formation, migration, and invasion miR‐421: Induces cell growth and apoptosis resistance miR‐663**:** Promotes cellular G1/S transition		57
	miR‐135a	IL‐17	Downregulation of miR‐135a associated with development of NPC following IL‐17 stimulation of pro‐inflammatory cytokine expression	Ovarian Cyst and Gallbladder Disease.	58
	hsa‐miR‐16, hsa‐miR‐34b and hsa‐miR‐449a, hsa‐miR‐34c, hsa‐miR‐101, hsa‐miR‐142, hsa‐miR‐504, hsa‐miR‐774.	hsa‐miR‐16: FGF2 hsa‐miR‐34b and hsa‐miR‐449a: LDHA hsa‐miR‐34c: MET hsa‐miR‐101: EZH2 hsa‐miR‐142: ZEB2 hsa‐miR‐504: NRF1 hsa‐miR‐774: TGF‐beta and cyclin B1.	hsa‐miR‐16: Inhibiting the NPC cell proliferation, migration, invasion, metastatic colonization hsa‐miR‐34b and hsa‐miR‐449a: Inhibiting the nasopharyngeal malignancy progression hsa‐miR‐34c: Suppressing the growth and metastasis of NPC tumor hsa‐miR‐101: Inhibiting the cellular processes, including cell di_erentiation, development and apoptosis. hsa‐miR‐142: Suppressing NPC cell proliferation, invasion and metastasis. hsa‐miR‐504: Inducing the radio‐resistance in NPC cells. hsa‐miR‐774: Promoting the nasopharyngeal malignant progression.		11
Adenoid cystic carcinoma	miR‐23b‐3p mir‐29a‐3p miR‐101‐3p miR‐181a‐5p MiR‐140‐5p	miR‐23b‐3p: PTEN mir‐29a‐3p: AKT2 miR‐101‐3p: Pim‐1, Survivin, Cyclin D1 and β‐catenin miR‐181a‐5p: LATS2 MiR‐140‐5p: Survivin	miR‐23b‐3p: Upregulation of angiogenesis and vascular permeability mir‐29a‐3p: High proliferation miR‐101‐3p: tumor suppressor role miR‐181a‐5p: oncogenic role	Affect invasion, proliferation, colony formation and cancer progression inliver, breast, prostate cancers	59, 60, 61, 62, 63
Melanoma	miR‐133a, miR‐199b, miR‐453, miR‐520f, miR‐521, miR‐551b, miR‐126, miR‐29c, miR‐506, miR‐507, and miR‐520d. miR‐190,	BRAF and NRAS genes, hyper‐activation of PI3K/AKT & WNT pathways, ApoE signaling, inactivation of p53 and alterations in CDK4/CDKN2A axis	Upregulation: miR‐133a, miR‐199b, miR‐453, miR‐520f, miR‐521, and miR‐551b, miR‐126, miR‐29c, miR‐506, miR‐507, and miR‐520d Downregulation: miR‐190, miR‐489 and miR‐527	Inducing malignant features	64
Neuroendocrine carcinoma	miRNA‐34a miRNA‐155 and miRNA‐21	miRNA‐155 interact with p53 protein as proapoptotic factor	Upregulation: oncogenic role	Improving metastases to lymph nodes Lung cancer	65
Nasopharyngeal carcinoma (NPC)	miR17‐5p miR93 and miR205 miR20a‐5p Let‐7d	miR17‐5p: p21 miR20a‐5p: NPAS2Let‐7d: AEG‐1	Upregulation: miR17‐5p increase proliferation by downregulating p21 protein, miR93 and miR205 increase cell growth and migration (oncogenic role) Down regulation: Let‐7dsuppressing proliferation and invasion and promotes apoptosis (tumor suppressing role)		66, 67

Abbreviation: miRNAs, microRNAs.

### 4.2 Sinonasal undifferentiated carcinoma (SNUC)

Sinonasal undifferentiated carcinoma is a rare and profoundly aggressive tumor with rapid development and a tendency to metastasize to multiple organs. It sometimes presents following radiotherapy of nasopharyngeal carcinoma or retinoblastoma. Immunohistochemically staining for cytokeratin or epithelial membrane antigen (EMA) is usually positive. The role of miR‐21 in tumorigenesis has been previously reported in lung, breast, stomach, prostate, colon, and pancreas cancers. An increased expression of miR‐21 is also found in undifferentiated sinonasal carcinoma suggesting its prognostic value in all these malignancies.^10^

### 4.3 Nasopharyngeal carcinoma

The nasopharyngeal carcinoma arises from epithelium of nasopharynx. Tobacco smoking, salt fish consumption, EBV infection, and vitamin C deficiency are the predisposing risk factors. It can manifest as neurologic symptoms due to metastasis to CNS. There are three histopathologic subtypes of nasopharyngeal carcinoma including keratinizing SCC, differentiated nonkeratinizing carcinoma, and undifferentiated nonkeratinizing carcinoma (mostly found in HPV‐positive cases).^68^ Although patients are managed by radiotherapy during the early stages, a combination of chemotherapy and radiation therapy is mostly preferred in advanced stages. Recent therapeutic approaches are based on targeted therapies such as epidermal growth factor receptor inhibitors (EGFR), angiogenesis inhibitors, and immunotherapy against EBV antigens.^69^ Also, some miRNAs with significantly altered expression are found to be involved in the pathogenesis of nasopharyngeal carcinoma (Table 7).

## 5 DISCUSSION

In this review, we presented altered expression of miRNAs in several premalignant and malignant head and neck tumors with epithelial origin. Different miRNAs play either as tumor suppressors that normally suppress cell proliferation, differentiation, and apoptosis or as oncogenes which are produced following gain‐of‐function alternations in proto‐oncogenes and thus contribute to tumorigenesis.^11^, ^70^ Hence, dysfunctional miRNAs exert a potential role as favorable biomarkers in the early diagnosis and prognosis of lesions or miRNA‐based target therapies.^52^ The miRNA‐based therapy is a twofold approach; first, replacement therapy in which synthetic/strategies miRNAs increase or restore the function of tumor suppressor miRNAs, and second, Strategies to inhibit oncogenic miRNAs which are mainly antisense oligonucleotides that silence oncogenic miRNAs, as well as spongious miRNAs, masking miRNAs, and small RNA inhibitors.^71^ Recently, small molecule‐miRNA associations (SMiR) approaches are found to inhibit miRNA biogenesis or target interaction. Although SMiR association network is expensive and time‐consuming, but it is exerted for identifying multiple cancer targets until now.^72^ However, unique miRNA has complicated function; one miRNA can target multiple genes and one gene can attach to various miRNAs, but this point is more accentuated when unique miRNA plays a double edge function. It is demonstrated when the expression of specific miRNA is altered in contrast to normal cell expression, they can play an oncogenic role or tumor suppressor function.^73^, ^74^ This can be generalized to a wide range of cancers.

In head and neck lesions like OSCC and OSMF, miR‐21 is an oncogenic miR that overexpressed in six solid cancers such as breast, lung, prostate, colon, oral cavity and acute myeloid leukemia. It seems miR‐21 overexpression reduces survival rate of carcinomas with a shorter disease‐free period. In this way, miR‐21 showed prognostic value.^16^, ^22^, ^41^ We describe more details in Tables 2 and 4. As well, miR‐31overexpresseed in lung cancer and play an oncogenic rolein Leukoplakia and OSMF. It detected in malignancies of lung cells and may play a vital role in oral cancer development and helps the cells to escape from apoptosis and chemotherapy.^15^, ^23^ It seems miR‐31 can present a more specific modulator role than miR‐21 during tumorigenesis process in oral epithelium because altered expression of miR‐21is observed in different tissues and can play a role in multiple cancer's molecular pathogenesis. The miR‐1246 increases invasion and metastasis by activation of the Wnt/β‐catenin pathway, and TGF‐β1‐induced type I collagen expression. This mechanism provides fibrogenesis in the oral cavity and also impacts liver, breast, and colon cancer progression^20^ (Table 2). It has been proposed that miR‐1246 can be a candidate for therapeutic approaches of submucosa fibrosis.^20^ Overexpression of miRNA‐146a/miRNA‐155 and miR‐150‐5p/miR‐222‐3pstimulate Th1 response in OLP in encounter to an unknown autoantigen and manifest the imbalance of Th1/Th2 cytokines like IFN‐γ production and also associate with tumor progression and lymph node metastasis. This cascade improves disease progression such as autoimmune disease (e.g., rheumatoid arthritis) (Table 3).^17^, ^27^ The miR‐146a overexpressed in oral cancer and enhance tumorigenesis and also it associate with downregulation of NUMB endocytic adapter protein (NUMB), the IL‐1 receptor associated with kinase 1 (IRAK1), and TNF receptor‐associated factor 6 (TRAF6) (Table 4).^41^

On the other hand, some miRNAs play a role as tumor suppressors. For example, miR‐203 downregulated N‐cadherin, vimentin, cell proliferation, and also increased CK19 and E‐cadherin proteins. The miR‐203 overexpression impedes arecoline‐induced epithelial‐mesenchymal transition and repressed proliferation‐related genes that reported in some cancers such as breast, pancreatic, ovarian, laryngeal, and hepatocellular cancers^21^ (Table 2). Thus, miR‐203 can prevents invasion and be a candidate for future therapeutic approaches, because influences cell cycle‐related genes, proliferation, and inflammation cascade, and also applied as a prognostic biomarker that affects survival rate in patients. One of the other miRs that are known as tumor suppressor factor are miR‐122 that has a critical role in inhibiting the tumorigenesis and angiogenesis in OLP and let‐7d that preventthe tumorigenesis process and downregulated in head and neck squamous cell carcinoma (HNSCC) (Tables 3, 4).^35^, ^39^ Also miR‐204 sole as tumor suppressor that involved in pathogenesis of relative common carcinoma of the maxillary sinus by targeting EphA7 gene.^55^ The dual regulatory role of miRNAs generalizes to different tissues and provides complication in a selection of specific microRNA for therapeutic strategies. It seems induce the expression of miRNA with tumor suppressor function or decrease the expression of miRNA with an oncogenic role can help us to use them as valuable therapeutic biomarkers. In addition to therapeutic approaches, oral miRNAs expression profile could serve as prognostic biomarkers by extracting them from body fluids (e.g., saliva, plasma, and blood) following a disease process or a therapeutic intervention.^52^, ^75^ Prognostic evaluations help us develop effective drugs or detect drug resistance.

## 6 CONCLUSION

The miRNAs are involved in a wide range of cancer pathogenesis and biogenesis and also can apply for early diagnosis and timely treatment that affect patient survival. The potential role of miRNAs as valuable biomarkers in diagnostic, prognostic, and potential therapeutic strategies in oral cancers can lead to appropriate early diagnosis of precancerous lesions, prevention of malignancy development, and improve disease prognosis. Although more investigations are needed to identify the best‐standardized protocol for miRNA isolation, altered expression miRNAs could be used for mentioned strategies in head and neck cancers as biomarkers with high sensitivity and specificity. A comprehensive strategy can help us to discover miRNAs with sufficient specificity and sensitivity for therapeutic approaches.

## AUTHOR CONTRIBUTIONS

**Alieh Farshbaf**: Data curation; investigation; visualization; writing – original draft; writing – review and editing. **Farnaz Mohajertehran**: Data curation; investigation; writing – original draft. **Amirhossein Sahebkar**: Investigation; visualization. **Yasaman Garmei**: Investigation; visualization. **Parisa Sabbagh**: Writing – original draft; writing – review and editing; writing – review and editing. **Nooshin Mohtasham**: Conceptualization; data curation; project administration; validation; visualization; writing – review and editing.

## CONFLICT OF INTEREST

The authors declare no conflict of interest. All authors have read and approved the final version of the manuscript, Dr. Nooshin Mohtasham as corresponding author had full access to all of the data in this study and takes complete responsibility for the integrity of the data and the accuracy of the data analysis.

## TRANSPARENCY STATEMENT

The lead author Nooshin Mohtasham affirms that this manuscript is an honest, accurate, and transparent account of the study being reported; that no important aspects of the study have been omitted; and that any discrepancies from the study as planned (and, if relevant, registered) have been explained.

## Data Availability

Data sharing not applicable to this article as no datasets were generated or analyzed during the current study.
